# Role of gamma-secretase in human umbilical-cord derived mesenchymal stem cell mediated suppression of NK cell cytotoxicity

**DOI:** 10.1186/s12964-014-0063-9

**Published:** 2014-09-30

**Authors:** Debanjana Chatterjee, Nicole Marquardt, Dejene Milkessa Tufa, Guillaume Beauclair, Hui Zhi Low, Tim Hatlapatka, Ralf Hass, Cornelia Kasper, Constantin von Kaisenberg, Reinhold Ernst Schmidt, Roland Jacobs

**Affiliations:** Department of Clinical Immunology and Rheumatology, Hannover Medical School, Carl-Neuberg-Str. 1, 30625 Hannover, Germany; Institute of Technical Chemistry, Leibniz University of Hannover, Hannover, Germany; Laboratory of Biochemistry and Tumor Biology, Clinic of Obstetrics and Gynecology, Hannover Medical School, Hannover, Germany; Department of Biotechnology, University of Natural Resources and Life Science, Vienna, Austria; Department of Obstetrics, Gynecology and Reproductive Medicine, Hannover Medical School, Hannover, Germany; Institute of Virology, Hannover Medical School, Hannover, Germany

**Keywords:** UC-MSC, NK cell cytotoxicity, Immunosuppression, IL-1β, Gamma-secretase

## Abstract

**Background:**

Mesenchymal stem cells (MSCs) are increasingly considered to be used as biological immunosuppressants in hematopoietic stem cell transplantation (HSCT). In the early reconstitution phase following HSCT, natural killer (NK) cells represent the major lymphocyte population in peripheral blood and display graft-vs-leukemia (GvL) effects. The functional interactions between NK cells and MSCs have the potential to influence the leukemia relapse rate after HSCT. Until date, MSC-NK cell interaction studies are largely focussed on bone marrow derived (BM)-MSCs. Umbilical cord derived (UC)-MSCs might be an alternative source of therapeutic MSCs. Thus, we studied the interaction of UC-MSCs with unstimulated allogeneic NK cells.

**Results:**

UC-MSCs could potently suppress NK cell cytotoxicity in overnight cultures via soluble factors. The main soluble immunosuppressant was identified as prostaglandin (PG)-E2. Maximal PGE2 release involved IL-1β priming of MSCs after close contact between the NK cells and UC-MSCs. Interestingly, blocking gamma-secretase activation alleviated the immunosuppression by controlling PGE2 production. IL-1 receptor activation and subsequent downstream signalling events were found to require gamma-secretase activity.

**Conclusion:**

Although the role of PGE2 in NK cell-MSC has been reported, the requirement of cell-cell contact for PGE2 induced immunosuppression remained unexplained. Our findings shed light on this puzzling observation and identify new players in the NK cell-MSC crosstalk.

**Electronic supplementary material:**

The online version of this article (doi:10.1186/s12964-014-0063-9) contains supplementary material, which is available to authorized users.

## Background

Mesenchymal stem cells (MSCs) are multipotent precursor cells that have the ability to differentiate mainly along chondrogenic, adipogenic and osteogenic lineages [1]. Although MSCs were originally isolated from bone marrow, it is now known that MSCs can be derived from various post-natal tissues like placenta, umbilical cord and cord blood, as well as adult tissues like tooth pulp, skin, adipose tissue, the nervous system and kidney [2,3]. MSCs can be defined as a heterogenous population of plastic-adherent cells with fibroblast-like morphology that express certain markers like CD90, CD73, and CD105, but are negative for surface markers of the hematopoietic lineage such as CD14, CD19, CD34, CD45, and HLA-DR [4].

MSCs have been reported to mediate profound immunosuppressive effects on T -, B -, NK cells, and dendritic cells [5]. MSCs are referred to as immunoprivileged as they possess a hypo-immunogenic profile due to lack of MHC class II molecules and classical co-stimulatory ligands as well as very low expression of MHC class I [6]. This immunoregulatory capacity together with their inherent non-immunogenicity makes them promising candidates for the use as biological immunosuppressants or tolerance inducers in alloimmune diseases like steroid resistant acute graft-versus-host-disease (GvHD) [7], organ transplantation or implantation of engineered tissues.

Natural killer (NK) cells are one of the main effectors of innate immunity due to their cytolytic activity against tumour cells and virus infected cells as well as by their ability to secrete various cytokines and chemokines [8]. NK cells comprise the main lymphocyte population in the early reconstitution phase after hematopoietic stem cell transplantation (HSCT) and mediate significant graft-vs-leukemia (GvL) effect [9,10] while MSCs are being increasingly used to treat GvHD following HSCT [11]. In therapeutic settings, the interaction of NK cells with MSCs might affect NK cell functions and the transplantation outcome. Clinical intervention to prevent any such adverse outcome of NK-MSC interaction requires precise understanding of the molecular mechanisms utilised by MSCs to suppress NK cells. Some work has been done to characterise the interaction of bone marrow derived MSCs (BM-MSCs) with NK cells [12–14]. However, interaction of umbilical cord derived MSCs (UC-MSCs) with NK cells is largely unexplored. Although effective, BM-MSCs are inherently afflicted with the issue of low proliferative potential and only very low numbers of MSCs can be isolated from bone marrow via painful invasive procedures. There is a constant hunt for better sources of MSCs for future clinical cell-based therapies. UC-MSCs have emerged as an ideal candidate owing to their non-invasive isolation procedure, superior proliferative potential and simple accessibility in large quantities [15]. Thus we chose to study UC-MSC–NK cell functional interplay.

This project was aimed at elucidating the underlying molecular mechanisms of UC-MSC mediated immune-modulatory effects on freshly isolated, human NK cells without the presence of any NK cell activating cytokine like IL-15 or IL-2 in the co-culture system. Previous work on BM-MSC-induced suppression of NK cell functions implicated soluble factors like indoleamine 2,3-dioxygenase (IDO), transforming growth factor (TGF)-β and prostaglandin (PG)-E2. PGE2 is the soluble immunosuppressant which is common to all reports till date [12,13,16]. However, the suppression of NK activation through PGE2 was reported to be contact dependent [13]. This indicated that the inhibition via PGE2 involved a more complex pathway that is yet to be understood. We also found that the UC-MSCs suppressed NK cell cytotoxicity mainly via PGE2 but the maximal release of PGE2 occurred only in contact cultures. Small amounts of interleukin (IL)-1β present in NK cells preparations were found to act on UC-MSCs to stimulate maximal PGE2 production. Additionally, we show that efficient IL-1 receptor (IL-1R) activation in MSCs require gamma-secretase enzyme activity. These findings further corroborate the recent reports on the identification of IL-1R as a novel gamma-secretase substrate [17].

## Results

### Phenotypic analysis and differentiation potential of UC-MSCs

**Figure 1:**
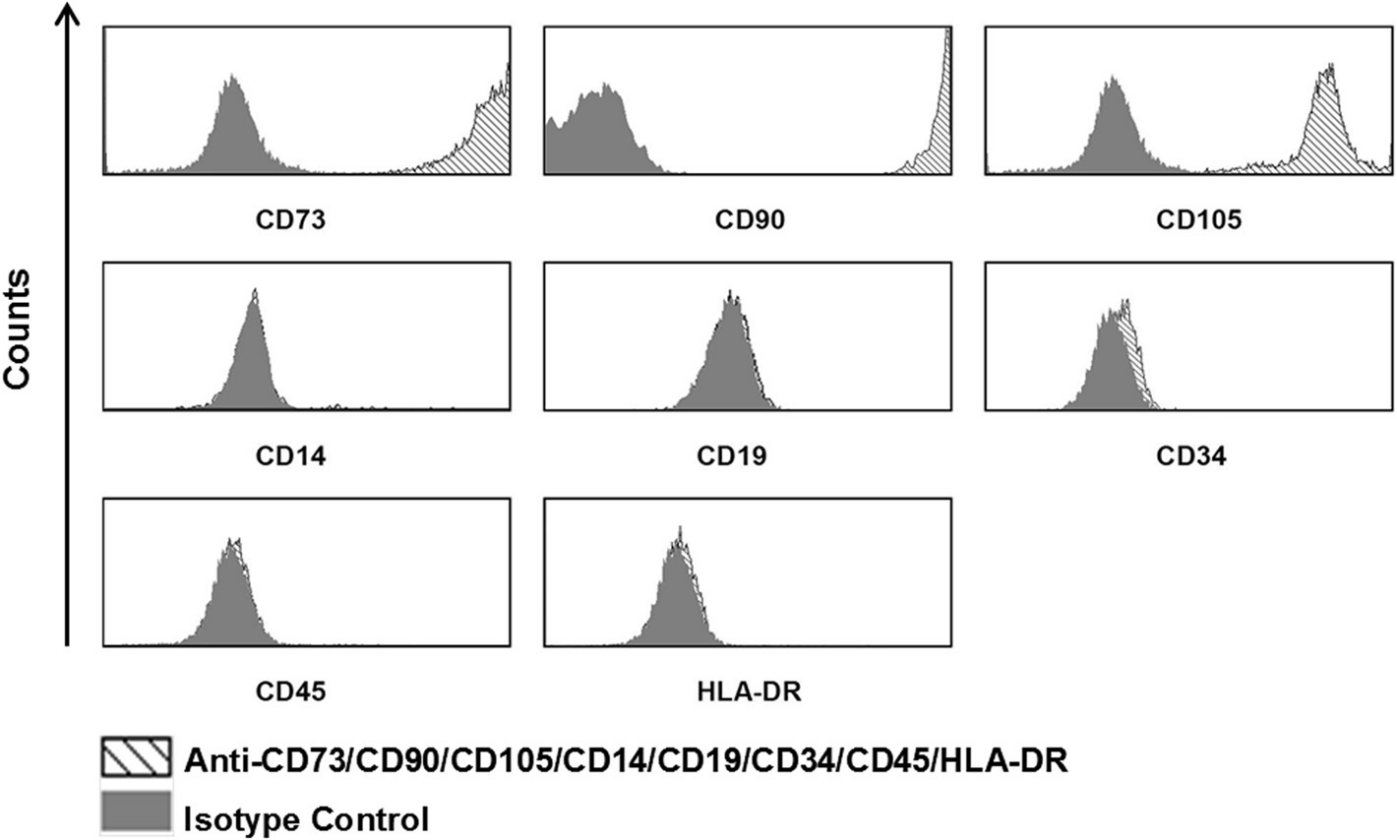
**Phenotype of UC-MSCs.** UC-MSCs were detached using accutase and stained with antibodies against CD14, CD19, CD34, CD45, CD73, CD90, CD105, and HLA-DR (shaded histograms) or isotype control antibodies (filled histograms).

The surface antigen expression patterns on the UC-MSCs were analysed using flow cytometry (Figure 1). CD73, CD90, and CD105 were highly expressed. MSCs lacked the expression of CD14, CD19, CD45, and HLA-DR on their cell surface. There was a very faint expression of CD34. The ability of the UC-MSCs to undergo chondrogenic, adipogenic and osteogenic differentiation was determined as described before (data not shown) [3]. Hence, the UC-MSCs fulfilled all the criteria recommended by the International Society for Cellular Therapy for identifying MSC [4].

### Effect of UC-MSCs on NK cell function and phenotype

**Figure 2:**
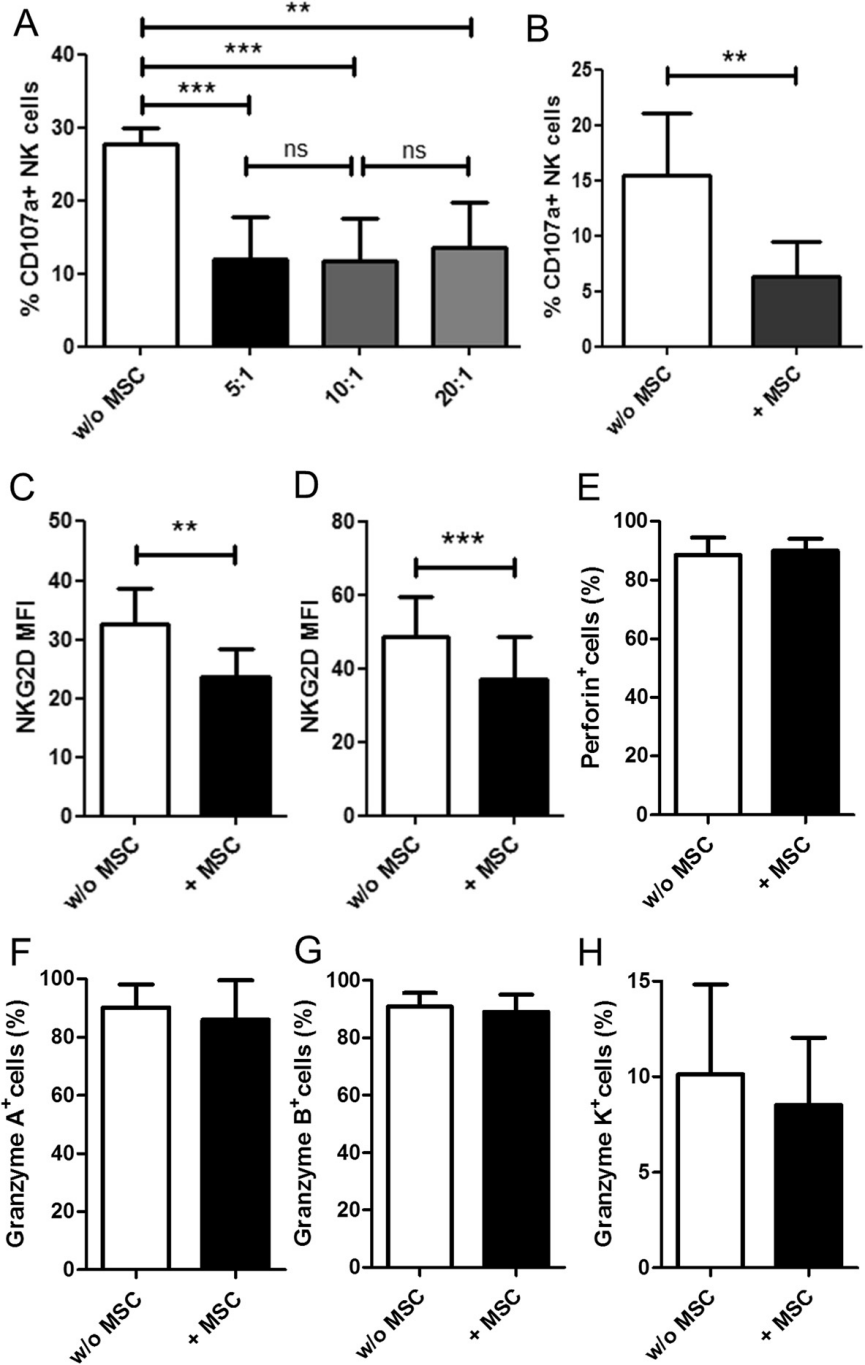
**Suppression of NK cell cytotoxicity by UC-MSCs. A**: NK cells were cultured alone or with MSCs at NK cell: MSC ratio of 5:1, 10:1, and 20:1. Following overnight co-culture, CD107a degranulation assay was performed with K562 target cells. The bar graphs represent the percentage of CD107a^+^ NK cells (n = 5) **B**: NK cells were cultured without MSCs or with MSCs at NK cell: MSC ratio of 10:1. CD107a degranulation assay was performed with K562 target cells (n = 5). Following overnight co-culture, CD107a expression on CD56 ^bright^ NK cells was analysed. The bar graphs represent the percentage of CD107a^+^ cells (n = 9). **C**, **D**: NK cells were cultured overnight with or without MSCs. NKG2D expression on the NK cells was analysed by flow cytometry. The bar graphs depict the mean fluorescence intensity (MFI) of NKG2D staining on CD56 ^dim^ (**C**; n = 9) and CD56 ^bright^ (**D**; n = 8) NK cells. **E**, **F**, **G**, **H**: NK cells were cultured overnight with or without MSCs. Perforin (**E**; n = 7), granzyme **A** (**F**; n = 7), granzyme **B** (**G**; n = 5), and granzyme K (**H**; n = 7) content of the NK cells was analysed by flow cytometry.

To examine the effect of MSCs on NK cells, freshly isolated, unstimulated NK cells were incubated with MSCs in direct contact at ratio 5:1, 10:1, and 20:1 for 16 hours. Following the co-culture, NK cells were harvested and exposed to K652 target cells to trigger NK cell degranulation. Surface expression of CD107a was analysed as a marker for degranulation. We found that NK cells pre-cultured with MSCs had significantly reduced degranulation capacity compared to NK cells cultured alone (Figure 2A). There was no significant difference in the suppression induced by different ratios of MSC during co-culture (Figure 2A). For all subsequent experiments, the NK: MSC ratio of 10: 1 was used. To confirm that the freshly isolated NK cells did not kill the MSCs during the co-culture, we performed chromium release assay with MSCs as target cells. We observed that the NK cells could efficiently lyse K562 cells but not the MSCs (Additional file 1: Figure S1). IL-15 pre-activated NK cells were better at killing the MSCs but the lysis was still less when compared to K562 (Additional file 1: Figure S1).

Following HSCT, CD56^bright^ NK cell subset is the major NK cell population in peripheral blood [9,18,19]. Our study aims to understand the fate of NK cells following MSC co-transfusion in HSCT settings. Therefore, we also specifically analysed the effect of MSCs on CD56^bright^ NK cells. We found that co-culturing with MSCs could significantly suppress K562-induced degranulation (Figure 2B) as well as IL-12 + IL-18 stimulated interferon-γ production by CD56^bright^ NK cells (Additional file 2: Figure S2). The observed suppression of NK cell cytotoxicity could have resulted from an altered expression of NK cells surface receptors which are crucial to NK cell activation. Using flow cytometry-based analysis, we looked for phenotypic changes on NK cells pre-cultured with MSCs compared to NK cells cultured alone. The panel of NK cell receptors analysed included CD16, CD27, CD62L, CD69, CD94, CD158, CD244, CD247, CD335, CD336, CD337, NKG2A, and NKG2D. Interestingly, the expression of all receptors tested was unaffected (Additional file 3: Figure S3) by MSCs, except for NKG2D. UC-MSCs significantly downregulated NKG2D receptor expression on both CD56^dim^ and CD56^bright^ NK cells (Figure 2C,D). We also examined if perforin or granzyme contents of NK cells were altered upon co-culturing with MSCs. Intracellular staining demonstrated that perforin and granzyme contents of NK cells cultured with MSCs were comparable to NK cells cultured alone (Figure 2E,F,G,H, Additional file 4: Figure S4).

### Mechanism of MSC-mediated suppression of NK cell cytotoxic functions

**Figure 3:**
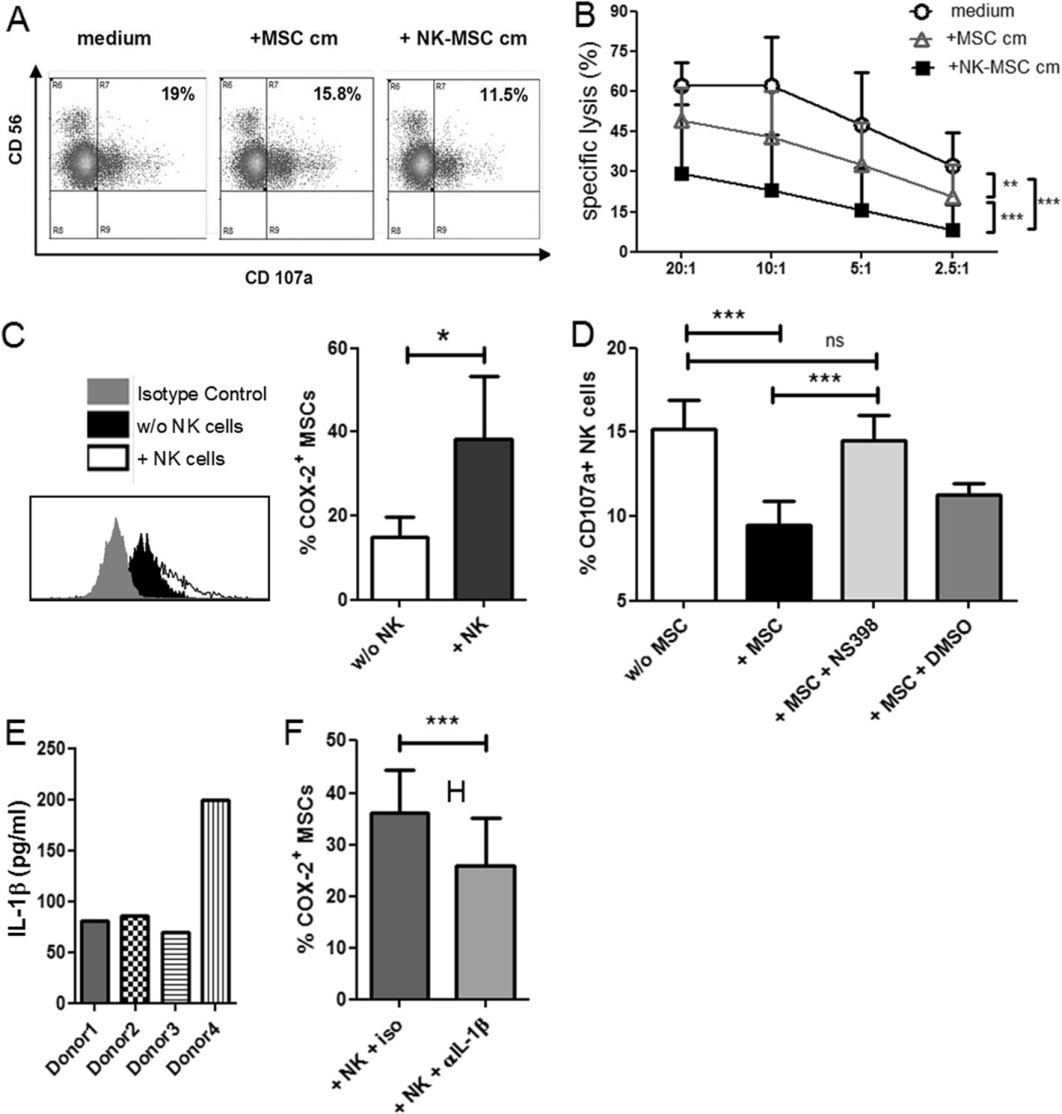
**Mechanism of MSC-mediated suppression of NK cell degranulation. A**: NK cells were cultured in normal medium or in MSC cm or in NK-MSC cm. CD107a degranulation assay was performed with K562 as target cells. Representative dot plots of the alterations in NK cell degranulation are shown (n = 3). **B**: NK cell-mediated cytotoxicity was determined by chromium release assay in different conditioned media (n = 3). **C**: UC-MSCs were cultured overnight with or without NK cells. Intracellular staining for COX-2 was performed and analysed by flow cytometry. One representative figure and bar graphs (n = 5) depicting COX-2 upregulation in UC-MSCs cultured with NK cells in comparison with COX-2 expression in “unprimed” UC-MSCs is shown. **D**: NK cells were cultured with or without MSCs or co-cultured in presence of NS-398 or DMSO. CD107a degranulation assay was performed with K562 target cells. The bars represent the effect of COX-2 inhibition on degranulative capacity of NK cells (n = 5). **E**: NK cells from four different donors were cultured for 16 hours and culture supernatants were collected. The bars represent the IL-1β levels measured in the supernatants (n = 4). **F**: MSCs were co-cultured with NK cells in presence of IL-1β neutralising antibody or matched isotype antibody. The bars illustrate the effect of IL-1β blocking on COX-2 expression (n = 4).

To verify the role of soluble factor(s) in MSC-induced suppression of NK cell cytotoxicity, we collected conditioned media (cm) from MSC cultures (MSC cm) and MSC-NK cell co-cultures (NK-MSC cm). NK cells were cultured in normal media or MSC cm or NK-MSC cm overnight and subsequently incubated with K562 target cells. Both MSC cm and NK-MSC cm were found to inhibit NK cell degranulation. However, NK-MSC cm was able to suppress NK cell degranulation more effectively than MSC cm (Figure 3A). These results were further corroborated by chromium release assays in which NK cells were cultured in normal media or MSC cm or NK-MSC cm overnight and subsequently incubated with radioactive chromium (^51^Cr) labelled K562 target cells. The extent of K562 lysis was determined by the ^51^Cr released into the culture supernatants. MSC cm suppressed NK cell cytotoxicity but to a significantly lower degree than NK-MSC cm (Figure 3B). This suggested that the suppressing soluble factor was present in lower amounts in MSC cm. Only when MSCs and NK cells came into contact, the soluble factor was released in larger amounts and resulted in enhanced suppression as seen with NK-MSC cm. MSC cm reduced cytotoxicity significantly, but not maximally, while the transwell system had no significant effect (Additional file 5: Figure S5). It has been reported by other groups [12,13] that indoleamine-2,3-dioxygenase (IDO) and cyclooxygenase (COX)-2 or transforming growth factor beta (TGF)-β produced by MSCs are responsible for suppression of NK cell cytotoxicity. We investigated if soluble end-products like prostaglandin (PG)-E2 or kynurenine (from COX-2 or IDO respectively) or TGF-β played any role in our system. Antibody mediated blocking of TGF-β and pharmacological inhibition of IDO by 1-MT did not result in increased degranulation (Additional file 6: Figure S6A,B). We found constitutive expression of COX-2 in UC-MSCs. Overnight co-culture of MSCs with NK cells resulted in significant upregulation of COX-2 (Figure 3C) in MSCs. However, no expression of COX-2 was observed in the co-cultured NK cells (Additional file 7: Figure S7). Blocking of COX-2 by a small-molecule inhibitor (NS-398) could significantly restore NK cell degranulation (Figure 3D). This indicated that PGE2 could be the major immunosuppressive factor released by MSCs inhibiting NK cell cytotoxicity. UC-MSCs, when cultured overnight in NK cell cm (NK cm), also upregulated COX-2 (data not shown). This indicated that some secretory molecules coming from NK cells could trigger COX-2 upregulation in UC-MSCs. We found that IL-1β is present in NK cm at low levels (Figure 3E). It is to be noted that the freshly isolated NK cells were producing IL-1β without prior exposure to MSCs. We hypothesized that this IL-1β could be responsible for the observed COX-2 upregulation in MSCs. To verify our hypothesis, we added IL-1β neutralising antibody in NK-MSC co-cultures. This resulted in significant inhibition of COX-2 upregulation (Figure 3F).

### Role of gamma-secretase in IL-1 signalling

**Figure 4:**
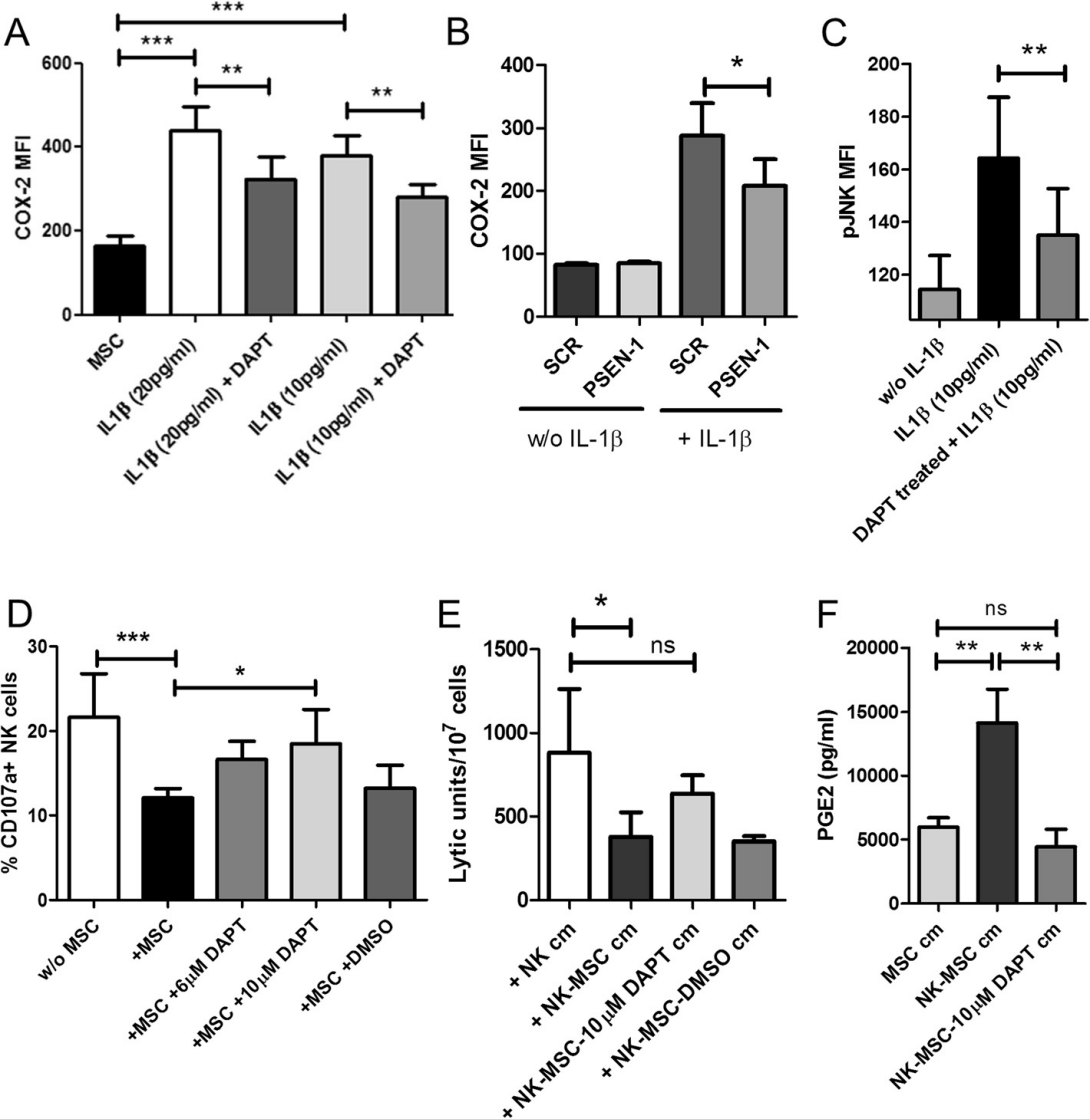
**Effect of gamma-secretase on IL-1 signalling and PGE2 content. A**: UC-MSCs were cultured in presence of different doses of IL-1β stimulation with or without DAPT (gamma-secretase inhibitor). UC-MSCs were detached using accutase and stained to analyse intracellular COX-2 expression (n = 5). **B**: Gamma-secretase activity in UC-MSCs was silenced using siRNA targeting a catalytic subunit of gamma-secretase (PSEN-1). Scrambled siRNA (SCR) was also introduced into UC-MSCs as control. After 24 hours, PSEN-1 or SCR siRNA treated MSCs were stimulated with IL-1β overnight. UC-MSCs were detached using accutase and stained to compare intracellular COX-2 expression with or without IL-1β stimulation (n = 3). **C**: UC-MSCs were stimulated with IL-1β for 40 minutes, with or without 2.5 hours pre-treatment with DAPT. MSCs were detached using accutase, immediately fixed, permeabilised and stained to analyse intracellular pJNK levels (n = 4). **D**: NK cells were cultured with or without MSCs or co-cultured in presence of 6 μM DAPT, 10 μM DAPT or DMSO. CD107a degranulation assay was performed with K562 target cells. The bars represent the effect of gamma-secretase inhibition on degranulative capacity of NK cells (n = 5). **E**: NK cells were cultured in NK cell conditioned media (NK cm) or in NK-MSC conditioned media (NK-MSC cm) or NK-MSC conditioned media where they were co-cultured in presence of 10 μM DAPT or DMSO (NK-MSC-10 μM DAPT cm or NK-MSC-DMSO cm respectively). Chromium release assay was performed with K562 target cells (n = 3). **F**: PGE2 concentration in MSC cm, NK-MSC cm or NK-MSC-10 μM DAPT cm as determined by competitive ELISA (n = 3).

IL-1β is known to act via IL-1 receptor present on target cells. IL-1 receptor (IL-1R) activity has been recently described to be gamma-secretase proteolysis dependent in HEK293T cells as well as mouse embryonic fibroblasts (MEFS) [17]. We examined if IL-1R signaling in human UC-MSCs depended on gamma-secretase activity. We cultured UC-MSCs with varying concentrations of IL-1β overnight in presence or absence of gamma-secretase inhibitor, DAPT. IL-1β was very efficient in upregulating COX-2 levels. However, the presence of DAPT significantly reduced COX-2 upregulation (Figure 4A). Pharmacological inhibitors can have a wide range of targets. To eliminate this possibility, we performed small interfering RNA (siRNA) mediated silencing of gamma-secretase activity in MSCs. We chose to knock-down Presenilin (PSEN)-1 because it is one of the catalytic subunits of gamma-secretase complex [20,21]. siRNA targeting PSEN-1 or control scrambled (SCR) siRNAs were introduced into the MSCs by electroporation. Knock-down of PSEN-1 mRNA was analysed after 24 hours by real-time PCR. PSEN-1 siRNA caused a 75% decrease in PSEN-1 mRNA compared to SCR siRNA (Additional file 8: Figure S8). In parallel with the assessment of mRNA knock-down, siRNA-treated MSCs were cultured for 16 hours in presence or absence of IL-1β. Similar to DAPT-mediated inhibition, PSEN-1 silencing significantly reduced COX-2 upregulation in response to IL-1β (Figure 4B). Introduction of PSEN-1 siRNA did not affect the basal COX-2 expression in MSCs. This clearly indicates the involvement of gamma-secretase in IL-1R signalling. Next, we tested if this effect of gamma-secretase blocking stemmed from impairment of IL-1R downstream signalling. We pre-treated MSCs with DAPT for 2.5 hours followed by IL-1β stimulation for 40 minutes. The DAPT pre-treated MSCs showed significant impairment in their ability to phosphorylate c-Jun N-terminal kinases (JNK) as mirrored in reduced phosphorylation of JNK following stimulation by IL-1β (Figure 4C). We further investigated if gamma-secretase influenced IL-1R signalling in our culture-setting. Pharmacological inhibition of gamma-secretase activity in NK-MSC co-cultures resulted in a restoration of NK cell degranulative capacity (Figure 4D). To establish that gamma-secretase blocking indeed affected the release of soluble suppressants, we performed the following experiment. Cell-free supernatants were collected from the NK-MSC co-cultures conducted in presence of DAPT or DMSO. They have been referred to as MSC-NK-DAPT conditioned medium (NK-MSC-DAPT cm) and MSC-NK-DMSO conditioned medium (NK-MSC-DMSO cm), respectively. NK cm and NK-MSC cm were also collected as controls. NK cells were cultured in these conditioned media followed by chromium release assay. NK-MSC cm caused a significant decline in lytic potential of NK cells. The cell-free co-culture supernatant generated in presence of gamma-secretase blocker, DAPT, had non-significant suppressive capacity. This indicated that NK-MSC-DAPT cm contained much lower quantity of the suppressant (Figure 4E). To directly test if the release of PGE2 was gamma-secretase dependent, we performed ELISA to analyse the concentration of PGE2 in MSC cm, NK-MSC cm, and NK-MSC-10 μM DAPT cm. We found that the amount of PGE2 increased significantly upon NK-MSC co-culture, but there was no increase in PGE2 if gamma-secretase activity was blocked (Figure 4F).

## Discussion

We observed that UC-MSCs were able to suppress NK cell effector functions in 16 hours of co-culture which is much shorter than the effective co-culture period described by other groups [12,13]. This discrepancy could be possibly explained by different culture conditions. The impairment of NK cell cytotoxicity towards K562 cells probably resulted from reduced degranulation. Interestingly, following co-culture with UC-MSCs, there was preferential downregulation of NKG2D on NK cells while no change was observed in the other activating receptors examined. NKG2D is one of the most prominent activating receptor on the NK cells [22]. The downregulation of NKG2D could be partially responsible for reduced NK cells degranulation towards K562 targets because this cell line expresses NKG2D activating ligand [23]. To understand if soluble factors have any role in the diminution of NK cell cytotoxicity, we compared cell-free supernatants from MSC cultures and NK-MSC contact cultures for their NK cells suppressive ability. It was evident from our experiments that soluble factors present in the media were responsible for the observed immunosuppression. NK-MSC cm was significantly superior to MSC cm in suppressing cytotoxic potential of NK cells indicating a need for a direct contact between the UC-MSCs and NK cells, for the maximal release of the cytotoxicity suppressant.

Soluble factors like TGF-β, kynurenine and PGE2 released by enzymatic action of COX-2 have been reported to be involved in inhibition of immune cell functions by BM-MSCs [12,13,24]. In our assays, antibody-mediated blocking of TGF-β and pharmacological inhibition of IDO via 1-methyl-tryptophan (MT) did not result in any restoration of the degranulative potential of NK cells. These differences could result from the use of unstimulated freshly isolated NK cells in our experiments. It is known that IDO is induced by IFN-γ in MSC [25], and freshly isolated NK cells did not produce considerable amount of IFN-γ (data not shown). We found that COX-2 was constitutively expressed in UC-MSCs. Blocking COX-2 resulted in significant restoration of NK cell degranulation suggesting that the soluble cytotoxicity suppressant is PGE2. IL-1β released by NK cells was measured to be in the range of 70-200 pg/ml. In a murine model of ulcerative colitis, IL-1β pre-treated MSCs were found to exhibit significantly higher therapeutic efficacy than untreated MSCs [26]. IL-1β is known to be a potent inducer of COX-2. Hence we neutralised IL-1β in our MSC-NK cell co-cultures and observed that blocking IL-1β indeed resulted in a decrease in COX-2 upregulation.

The induction of COX-2 is dependent on IL-1β release from NK cells. Hence, IL-1β produced by NK cells in transwell co-cultures should also induce COX-2 and PGE2 production. However, no suppression was observed in transwell co-cultures. This can possibly be explained by the low levels of IL-1β produced by NK cells, making direct cell-cell contact a pre-requisite for attaining effective concentrations for bioactivity. In transwell co-cultures, the low IL-1β concentrations appear insufficient to upregulate COX-2 in MSCs. It is important to note that our co-culture duration is just 16 hours. In conditioned media collected from NK cultures (NK cm) after 16 hours, IL-1β has probably reached effective concentrations as implicated by COX-2 upregulation in MSCs exposed to NK cm.

Previous work has demonstrated that gamma-secretase acts on IL-1R and controls the downstream signalling following receptor activation by ligand binding [17]. We hypothesized that the IL-1R signalling in MSCs could also be gamma-secretase dependent. We inhibited gamma-secretase enzyme activity using pharmacological inhibitors or siRNA-mediated knockdown. We found that the inhibition of gamma-secretase significantly reduced COX-2 upregulation in MSCs in response to IL-1R activation by IL-1β. We could also demonstrate that gamma-secretase inhibition also affected JNK phosphorylation down-stream of IL-1R activation. Therefore, we could demonstrate that IL-1R signalling in MSCs was dependent on gamma-secretase activity. This dependence was also mirrored in the drastic mitigation of PGE2 release under conditions of gamma-secretase inhibition. Finally, pharmacological inhibition of gamma-secretase during NK cell-MSC co-cultures showed significant restoration of NK cell degranulation.

## Conclusion

Use of MSCs for cell therapy is on the rise, and BM-MSCs have been shown to be advantageous for the treatment and prevention of GvHD as well as facilitation of engraftment following HSCT in several clinical trials [27]. The mechanistic insights obtained from in-vitro studies indicate that the anti-inflammatory effects of MSCs, that ameliorate complications following HSCT, stem from suppression of aberrant T-cell proliferation [25]. Following HSCT, the first lymphocyte population to reconstitute are NK cells, which contribute to improved engraftment, reduced rates of leukemia relapse [28] and decreased GvHD. MSCs co-administered with HSC to suppress T-cells are also likely to suppress NK cell functions, thereby eliminating their beneficial effects.

We found that UC-MSCs indeed suppress NK cell cytotoxicity in overnight contact cultures. The suppression is mediated by the soluble suppressant PGE2. However, the maximal release of PGE2 required a cross-talk between NK cells and MSCs involving IL-1β/IL-1R signalling. The low IL-1β levels are probably more effective within the immunological synapse formed in contact cultures. This could explain the contact dependence of observed suppression. We also show that the IL-1R signalling is dependent on gamma-secretase enzyme activity. Finally, pharmacological inhibition of gamma-secretase significantly alleviated MSC-induced suppression. We need to further investigate if these immunosuppressive mechanisms also apply to MSCs derived from other sources. Identification of common molecular pathways utilised by MSCs, irrespective of the source, could have significant clinical implications, especially in case of HSCT.

## Materials and methods

### Isolation and culture of UC-MSCs

This study was approved on 26th February, 2009 by the Institutional Review Board (Project No. 3037) in an extended permission # 443. After obtaining written consent, MSCs were isolated from at least four different human umbilical cords obtained from full-term infants (38-40 weeks) by explant culture, expanded and cryopreserved until the start of the experiment as described elsewhere [3,29]. αMEM supplemented with 10% human serum and 50μg/ml streptomycin, 50U/ml penicillin, 1mM glutamine and 0.5mM sodium pyruvate (Biochrom, Berlin, Germany) was used to culture UC-MSCs for all subsequent experiments.

### Isolation of peripheral blood mononuclear cells (PBMC) and NK cells

Heparinised blood samples were obtained from healthy consenting donors and diluted with an equal volume of phosphate buffered saline (PBS). PBMC were separated by centrifugation over a ficoll-hypaque gradient and stained with anti-CD56 and CD3 antibodies. NK cells were sorted using MoFlo Cell Sorter (Beckman Coulter) at the MHH sorting facility. NK cells were defined as CD56^+^CD3^−^ lymphocytes.

### Monoclonal antibodies (mAbs)

The anti-human mAbs have been used: CD3 PE (from Beckman Coulter), CD3 PerCP, CD11b PE, CD14 APC, CD56 PE, CD56 APC, CD73 PE, CD107a FITC, Granzyme A FITC, Perforin FITC, pJNK PE (from BD Biosciences), HLA-DR FITC (Dako), CD44 FITC, CD105 FITC, Granzyme B PE, Granzyme K Alexa647 (Immunotools), CD90 APC, COX2 FITC (Cayman chemicals). Each flow cytometric analysis was controlled with isotype-matched mAbs. All flow cytometry-based experiments were performed on FACS Calibur using Cell-Quest Pro Software. Offline data analysis was done on Summit 5.1 software.

### Cell culture

UC-MSCs were seeded in 24- or 48-well plates and allowed to adhere. After 24 hours, freshly isolated, FACS-sorted NK cells from unrelated donors were added at a MSC:NK-ratio of 1:10. Following 16 hours of co-culture, the NK cells were removed from the adherent MSCs by pipetting for phenotyping or were tested for cytokine producing ability by intracellular staining or for cytotoxic potential against K562 leukemic cells using CD107a degranulation or chromium release assay. NK cells cultured without MSCs were used as controls. Cell-free supernatants from the NK-MSC co-cultures or MSC cultures were frozen for further analysis as MSC-NK-conditioned medium (NK-MSC cm) or MSC conditioned media (MSC cm), respectively. For determining the influence of conditioned media, NK cells from the unrelated donors were cultured for 16 hours in the cell-free supernatants generated as mentioned above. To determine the role of gamma-secretase activation, N-[N-(3,5-Difluorophenacetyl)-L-alanyl]-S-phenylglycine t-butyl ester (DAPT) (Sigma-Aldrich, Germany), a gamma-secretase inhibitor, was added to the co-cultures at 6 μM or 10 μM concentrations. DMSO was used as vehicle control. 5 μM NS-398 was added to the co-cultures to block the action of cyclooxygenase (COX)-2. IL-1β neutralising antibody or the matched isotype control antibody was added to the co-cultures at a concentration of 10 ng/ml.

### Detection of intracellular proteins

For detection of perforins and granzymes, after surface staining, cells were fixed with 4% paraformaldehyde (Merck) for 10 min. Subsequently, the cells were perforated in 0.1% saponin buffer (PBS supplemented with 0.1% Saponin (Riedel-de-Haën) and 0.01M HEPES (Roth) and fluorochrome-tagged monoclonal antibodies (mAbs) were added. After 30 minutes of incubation and three washes, cells were analysed by flow cytometry.

For detection of COX-2, the MSCs were detached by Accutase (Life technologies) treatment at 37°C for 5 min and fixed with 4% paraformaldehyde followed by 30 minute incubation with anti-COX-2 mAb in saponin buffer. The cells were then washed and analysed by flow cytometry.

For detection of pJNK, MSCs were stimulated with IL-1β for 40 minutes and washed once with PBS. Detachment of MSCs using Accutase was immediately followed by fixation using 1X BD Lyse/Fix Buffer. The permeabilisation and intracellular staining for pJNK was performed according to manufacturer’s protocol.

### CD107a degranulation assay

NK cells cultured with or without MSCs were incubated with K562 target cells in presence of anti-CD107a mAb at an Effector:Target (E:T) ratio of 10:1. Monensin (BD Biosciences) was added after 1 hour at a final concentration of 6 μg/ml and the incubation was continued for 3 hours. The cells were then analysed for the surface-expression of CD107a by flow cytometry.

### Cytotoxicity assay against K562 target cells

Cytotoxicity of NK cells, cultured under different conditions e.g in presence/absence of MSCs or in the presence/absence of conditioned media, was determined by 4 hour chromium release assay against K562 target cells following the protocol as described elsewhere [30]. Briefly, K562 cells were labelled with radioactive ^51^Cr by incubating the cells with 3 MBq Na^51^CrO_4_ for 1h at 37°C. The cells were washed twice and plated in triplicates in V-bottom 96-well plates. Effector: Target ratios of 20:1, 10:1, 5:1 and 2.5:1 were used. NK cell mediated lysis of targets results in the release of ^51^Cr into the supernatant. Following the 4 hour incubation, the plates were centrifuged. 25 μl cell-free supernatant was collected from each well, and the ^51^Cr release was measured by a gamma counter. Lytic units (LU_20_/10^7^ cells) of the assays were calculated according to the methods established by Bryant *et al.* [31].

### Cytokine bead array

The amount of IL-1β present in the culture supernatants of NK cells was measured using the cytometric bead array kit (BD Biosciences) in combination with human IL-1β Flex set according to the manufacturer’s protocol. Briefly, fluorescently labelled beads (bead position B4) were mixed with known standards or test samples followed by incubation with PE-conjugated detection antibodies. The samples were washed, measured on FACS Canto II and analysed using the BD CBA analysis software.

### Prostaglandin(PG)-E2 ELISA

PGE2 was measured in culture supernatants by competitive enzyme-linked immunosorbent assay (ELISA) technique using a commercially available ELISA kit (Enzo Life Sciences), according to the manufacturer’s protocol. Concentrations were calculated by comparison with known PGE2 standards using a 5 parameter logistic curve fitting program.

### siRNA transfections

The following small interfering RNA (siRNA) were obtained from Dharmacon, Thermo Scientific: ON-TARGETplus Non-targeting Control Pool (D-001810-10-05), ON-TARGETplus PSEN1; Set of 4 (LQ-004998-00-0002). The four individual PSEN1 targeting siRNAs were mixed (i.e. 37.5 pmol each) before use. Transfection with siRNAs was performed using the Neon transfection system (Invitrogen) at 1350 V, 10 ms, 4 pulses; according to the manufacturer’s instructions. siRNAs were microporated at the concentration of 150 pmol into 8×10^4^ cells.

### Real-time PCR

Total RNA was isolated from siRNA-treated UC-MSCs using RNAeasy Micro Kit (Qiagen), according to manufacturer’s protocol. cDNA was prepared using a commercially available reverse transcription kit (Applied Biosystems; Cat. No: 4368814). Expression of PSEN-1 mRNA relative to β-actin was analyzed using semi-quantitative PCR. All experiments were performed in triplicates. Fold change in PSEN-1 mRNA expression was calculated using the 2^-ΔΔCT^ method. The following primers were used: PSEN-1 primer pair (SantaCruz Biotechnology, Inc.; Cat. No: sc-36312-PR) and β-actin quantitect primers (Qiagen.; Cat. No: QT00095431).

### Statistical analyses

Paired two-tailed *t*-tests or ANOVA with Bonferroni post-test were performed using GRAPHPAD PRISM V5.00 Software. Levels of significance are shown as *p*-values (* *p* < 0.05, ** *p* < 0.01, *** *p* < 0.001). Bar graphs represent mean +/- standard deviation (SD).

## Additional files

Additional file 1: Figure S1.
**Specific lysis of UC-MSCs by NK cells.** MSCs or K562 (control) were used as target (T) cells. Freshly isolated, unstimulated NK cells or IL-15-preactivated NK cells were used as effector cells. When MSCs were used as targets, MSCs were seeded in flat-bottom 96 well plates and cultured overnight to obtain adherent MSCs, prior to addition of NK cells. Effector (E) cells were subsequently added to the targets and chromium release assay was performed (n = 3). Additional file 2: Figure S2.
**Effect of UC-MSCs on IFN-γ production by CD56** ^**bright**^ **NK cells.** NK cells were cultured with or without MSCs. The NK cells were harvested and stimulated with IL-12 and IL-18. Brefeldin A was added after 1 hour of culture. At the end of 4 hours of stimulation, the cells were intracellularly stained for IFN-γ, and analysed by flow cytometry (n = 6). Additional file 3: Figure S3.
**Effect of UC-MSCs on NK cell phenotype.** NK cells were cultured with or without MSCs for 16 hours. The cells were surface stained and the expression of the indicated receptors was analysed by FACS. All experiments were repeated at least three times. Additional file 4: Figure S4.
**Effect of MSCs on perforin and granzyme content of NK cells.** NK cells were cultured overnight with or without MSCs. Perforin (n = 7), granzyme A (n = 7), granzyme B (n = 5), and granzyme K (n = 7) content of the NK cells was analysed by flow cytometry. Representative dot plots, depicting the staining for perforin **(A)** and granzymes **(B, C, D)**, are shown. Additional file 5: Figure S5.
**Impact of UC-MSCs on NK cell cytotoxicity in transwell settings.** NK cells were cultured without MSCs or with MSCs separated by transwell inserts. MSCs were seeded in the lower well before NK cells were added in transwell inserts. Chromium release assay was then performed to assess the cytotoxic potential of the NK cells using K562 as target cells. Additional file 6: Figure S6.
**Effect of blocking TGF-β and IDO on UC-MSC mediated suppression.** **A**: NK cells were cultured overnight with or without MSCs or with MSCs in presence of TGF-β blocking antibody. The NK cells were harvested and CD107a degranulation assay was performed with K562 as target cells (n = 4). **B**: NK cells were cultured overnight with or without MSCs or with MSCs in presence of 1-MT. The NK cells were harvested and CD107a degranulation assay was performed with K562 as target cells (n = 4). Additional file 7: Figure S7.
**Lack of COX-2 expression in NK cells cultured with MSCs.** NK cells cultured with MSCs were harvested. The cells were surface stained, followed by intracellular staining with anti-COX-2 antibody or matching isotype control antibody. Representative dot plots, depicting the lack of COX-2 expression in NK cells, are shown (n = 4). Additional file 8: Figure S8.
**siRNA mediated knock-down of PSEN-1.** siRNA targeting PSEN-1 or scrambled controls (SCR) were introduced into the UC-MSCs using electroporation. 24 hours later, total RNA was isolated from siRNA-treated UC-MSCs, and cDNA was prepared. Expression of PSEN-1 mRNA relative to β-actin was analyzed using semi-quantitative PCR. All experiments were performed in triplicates. The bar-graphs depict the fold change in PSEN-1 mRNA expression as calculated using the 2^-ΔΔCT^ method (n = 3).
