# Supplementary figures and images for: Role of gamma-secretase in human umbilical-cord derived mesenchymal stem cell mediated suppression of NK cell cytotoxicity
Source: Cell Commun Signal. 2014 Sep 30;12:63. doi: 10.1186/s12964-014-0063-9 (PMC4195898; doi:10.1186/s12964-014-0063-9)

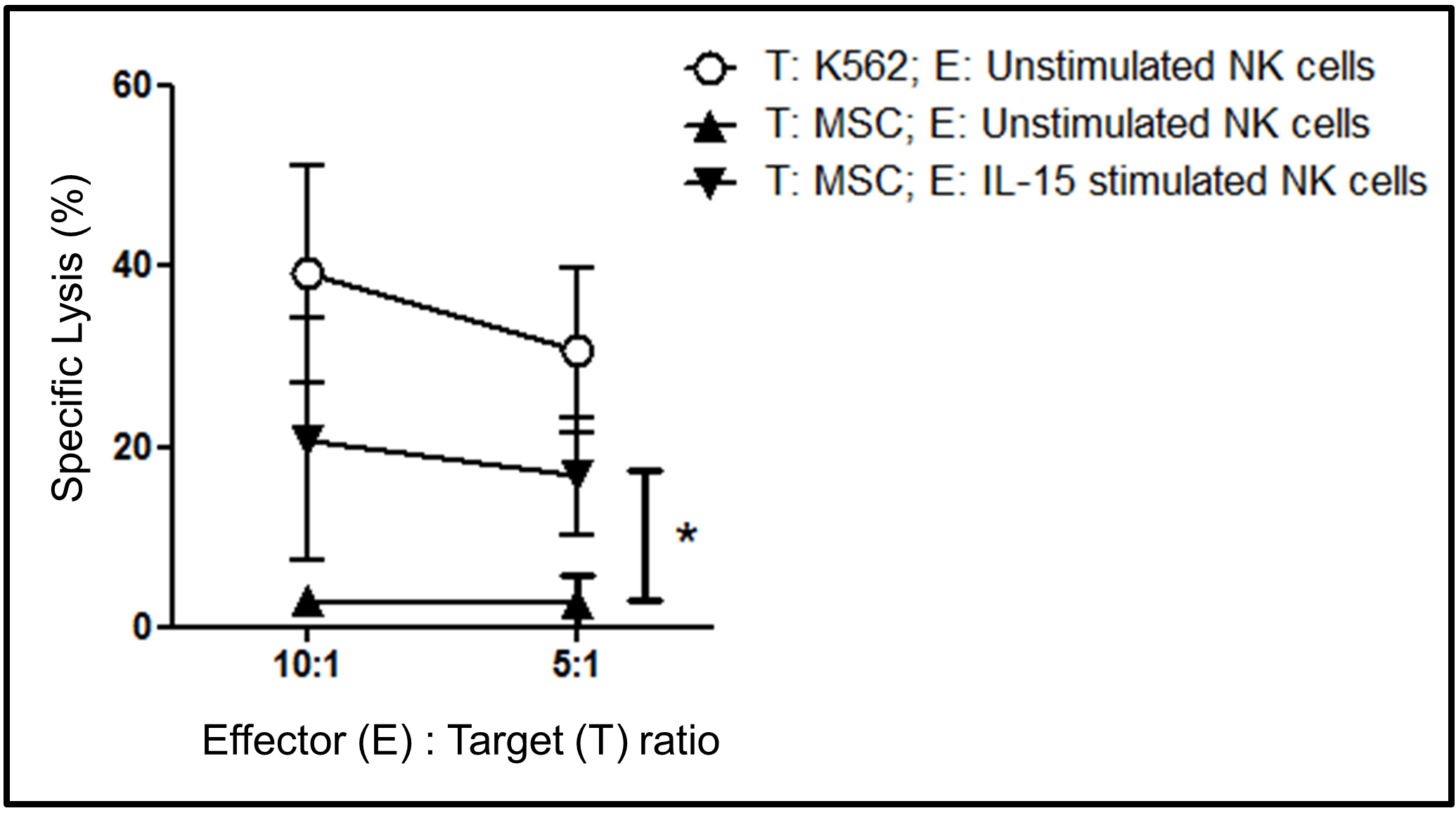

Supplement: Additional file 1: Figure S1. — Specific lysis of UC-MSCs by NK cells. MSCs or K562 (control) were used as target (T) cells. Freshly isolated, unstimulated NK cells or IL-15-preactivated NK cells were used as effector cells. When MSCs were used as targets, MSCs were seeded in flat-bottom 96 well plates and cultured overnight to obtain adherent MSCs, prior to addition of NK cells. Effector (E) cells were subsequently added to the targets and chromium release assay was performed (n = 3). [file 12964_2014_63_MOESM1_ESM.tiff]

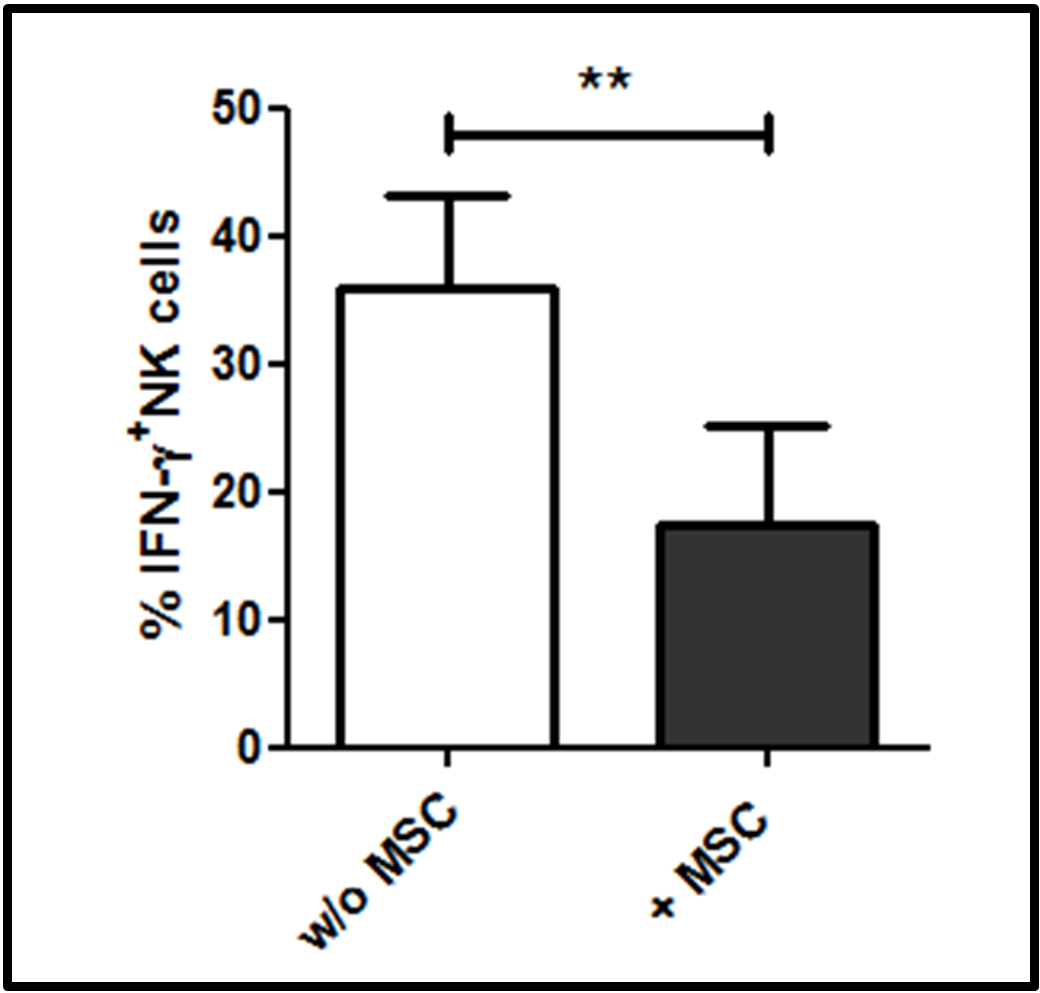

Supplement: Additional file 2: Figure S2. — Effect of UC-MSCs on IFN-γ production by CD56 bright NK cells. NK cells were cultured with or without MSCs. The NK cells were harvested and stimulated with IL-12 and IL-18. Brefeldin A was added after 1 hour of culture. At the end of 4 hours of stimulation, the cells were intracellularly stained for IFN-γ, and analysed by flow cytometry (n = 6). [file 12964_2014_63_MOESM2_ESM.tiff]

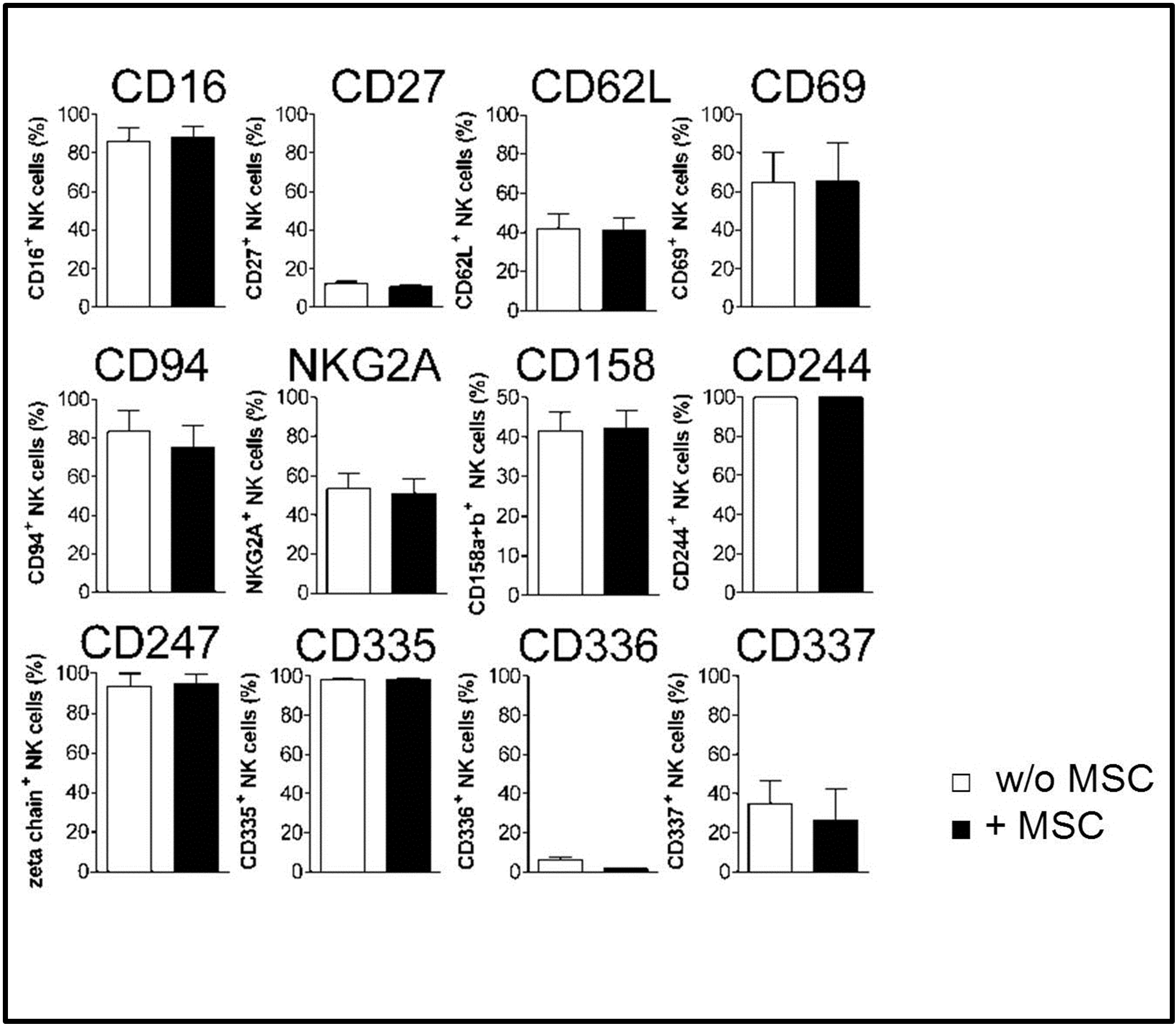

Supplement: Additional file 3: Figure S3. — Effect of UC-MSCs on NK cell phenotype. NK cells were cultured with or without MSCs for 16 hours. The cells were surface stained and the expression of the indicated receptors was analysed by FACS. All experiments were repeated at least three times. [file 12964_2014_63_MOESM3_ESM.tiff]

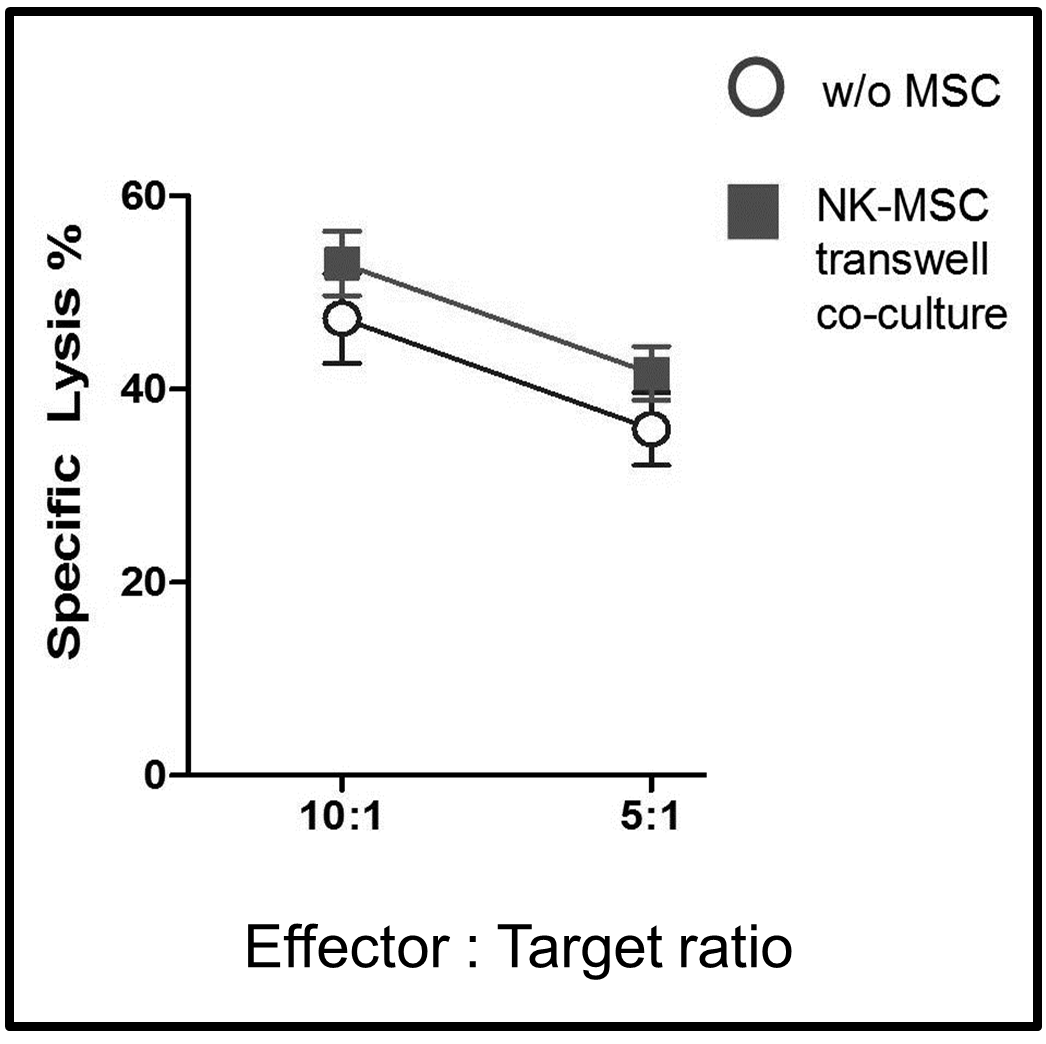

Supplement: Additional file 5: Figure S5. — Impact of UC-MSCs on NK cell cytotoxicity in transwell settings. NK cells were cultured without MSCs or with MSCs separated by transwell inserts. MSCs were seeded in the lower well before NK cells were added in transwell inserts. Chromium release assay was then performed to assess the cytotoxic potential of the NK cells using K562 as target cells. [file 12964_2014_63_MOESM5_ESM.tiff]

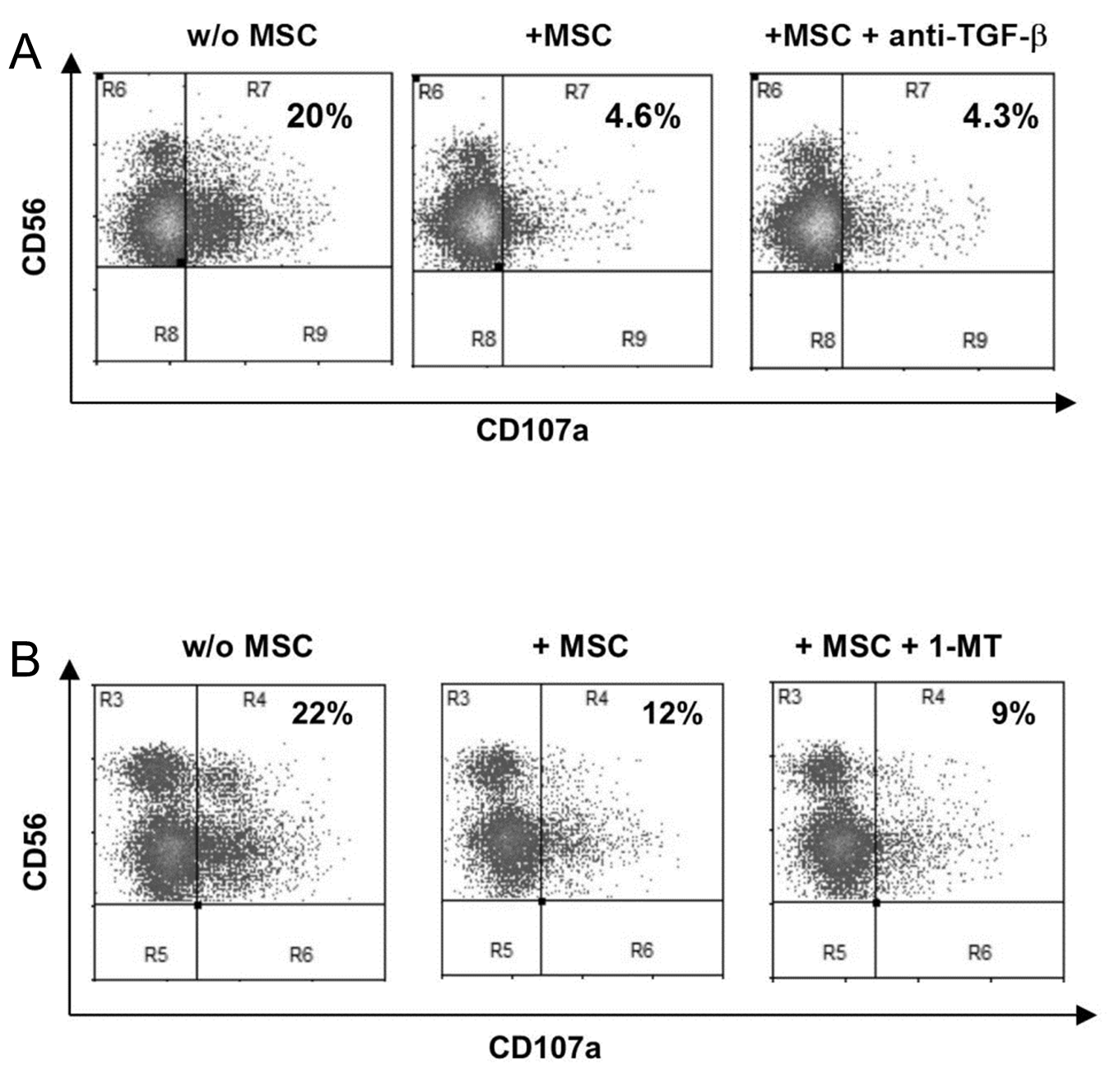

Supplement: Additional file 6: Figure S6. — Effect of blocking TGF-β and IDO on UC-MSC mediated suppression. A: NK cells were cultured overnight with or without MSCs or with MSCs in presence of TGF-β blocking antibody. The NK cells were harvested and CD107a degranulation assay was performed with K562 as target cells (n = 4). B: NK cells were cultured overnight with or without MSCs or with MSCs in presence of 1-MT. The NK cells were harvested and CD107a degranulation assay was performed with K562 as target cells (n = 4). [file 12964_2014_63_MOESM6_ESM.tiff]

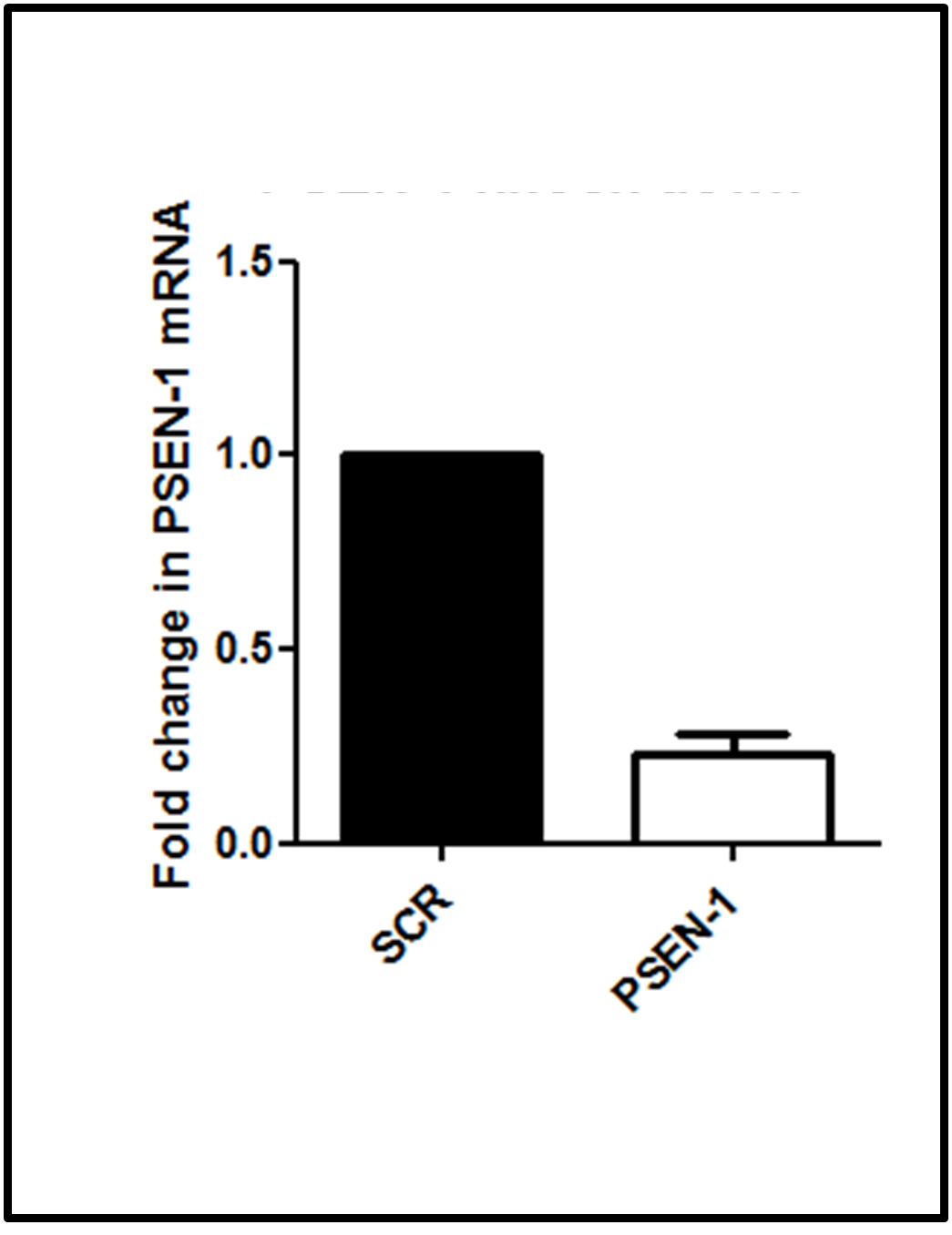

Supplement: Additional file 8: Figure S8. — siRNA mediated knock-down of PSEN-1. siRNA targeting PSEN-1 or scrambled controls (SCR) were introduced into the UC-MSCs using electroporation. 24 hours later, total RNA was isolated from siRNA-treated UC-MSCs, and cDNA was prepared. Expression of PSEN-1 mRNA relative to β-actin was analyzed using semi-quantitative PCR. All experiments were performed in triplicates. The bar-graphs depict the fold change in PSEN-1 mRNA expression as calculated using the 2-ΔΔCT method (n = 3). [file 12964_2014_63_MOESM8_ESM.tiff]
